# Push-Out Bond Strength of Experimental Apatite Calcium Phosphate Based Coated Gutta-Percha

**DOI:** 10.1155/2018/1731857

**Published:** 2018-08-01

**Authors:** Afaf Y. Al-Haddad, Muralithran G. Kutty, Zeti Adura Che Ab Aziz

**Affiliations:** ^1^Department of Dental Materials and Equipment, Faculty of Dentistry, Mahsa University, 42610 Selangor, Malaysia; ^2^Department of Restorative Dentistry, Faculty of Dentistry, University of Malaya, 50603 Kuala Lumpur, Malaysia

## Abstract

**Objectives:**

To evaluate the push-out bond strength of experimental apatite calcium phosphate coated gutta-percha (HAGP) compared to different commercially available coated gutta-percha root obturation points.

**Methods:**

Extracted teeth were selected and instrumented using ProTaper rotary files. The canals were assigned into five equal groups and obturated using matching single cone technique as follows: EndoREZ cones and EndoREZ sealer, Bioceramic Endosequence gutta-percha (BCGP) with Endosequence BC sealer, Active GP with Endosequence BC sealer (ActiV GP), conventional GP with Endosequence BC sealer, and HAGP with Endosequence BC sealer. Each root was sectioned transversally at the thickness of 1±0.1 mm to obtain 5 sections (n=25 per group). The specimens were subjected to push-out test using a Universal Test Machine at a loading speed of 0.5 mm/ min. Failure modes after push-out test was examined under stereomicroscope and the push-out data were analyzed using ANOVA and the post hoc Dunnett T3 test (p = 0.05).

**Results:**

The highest mean bond strength was yielded by HAGP followed by BCGP, ActiV GP, conventional GP, and EndoREZ. There were significant differences between EndoREZ and all other groups (p<0.001). The prominent failure mode of HAGP was mixed mode, whereas EndoREZ exhibited adhesive failure mode. Conventional GP, ActiV GP, and BCGP showed cohesive failure mode.

**Conclusion:**

HAGP showed promising results to be used as root canal filling material in combination with bioceramic sealer.

## 1. Introduction

One of the desirable properties of the root canal obturation material is its ability to adhere to root dentine [1]. The adhesion influences both sealing ability and root strength [2, 3]. Good adhesion eliminates any gap that would allow penetration of fluids between the filling and the dentinal wall. It also resists dislodgement of fillings during subsequent manipulation such as postspace preparation [4].

Gutta-percha (GP) is the standard root canal obturation material that has many advantages such as biocompatibility, nonstaining, and radiopaque. It can also be easily removed from the root canal when necessary [5]. However, GP cannot spontaneously bond to root dentine [6] or to sealers, leading to microleakage resultant of voids and gaps between GP and sealer [7]. In order to obtain bonding of GP to sealers, a concept of coating the GP with a material that is similar to the components of the root canal sealer has been introduced. This is to improve its bonding to the sealer and forming a single unit that bond to root dentine which is called “monoblock”.

EndoREZ® (Ultradent Products Inc., USA) is a root canal obturation system composed of urethane dimethacrylate resin based sealer and methacrylate resin-coated GP cones. The manufacturer claimed that EndoREZ cones and the sealer could form a monoblock within the root canal space. However, EndoREZ system was found to have poor bond strength to root dentine due to the polymerization shrinkage of its sealer [8, 9]. Further, the presence of interfacial gaps between the sealer and root dentine as well as the sealer and core material has also been shown [10].

Glass ionomer-impregnated and coated GP (ActiV GP, Brasseler, USA) is another approach to address the poor adhesive property of GP. It is produced to be used with glass ionomer-based sealer. The bond strength of ActiV GP was higher compared to methacrylate-based obturation systems (EndoREZ and Resilon). However, it was lesser than that of AH plus/GP [9].

Endosequence BC coated GP (Brasseler, USA) is one of the latest innovative materials composed of Endosquence BC sealer (Brasseler, USA) coating and impregnated GP cones. The Endosequence BC sealer components consisted of calcium phosphate, calcium silicate, calcium hydroxide, and zirconium oxide. Endosequence BC coated GP was used without sealer and it was found to have the significantly lowest mean bond strength compared with AH plus and conventional GP [11]. However, no current data is available about BCGP bond strength to dentine when combined with Endosequence BC sealer.

Recently, a novel apatite calcium phosphate coated gutta-percha (HAGP) was produced to enhance the adhesion of GP cones to root sealers, and subsequently to root dentine [12]. Theoretically, using bioactive hydroxyapatite can lead to the deposition of inorganic particles on the root surface during the degradation process, thereby increasing sealing ability and inducing the growth of crystals on the material surfaces [13]. Moreover, the similarity of components of hydroxyapatite to the inorganic components of root dentine could expand the use of HAGP to most of the root sealers available in the market. The aim of this study is to evaluate and compare the push-out bond strength of the novel HAGP to different types of commercially available coated GP root obturation materials.

## 2. Materials and Methods

### 2.1. Specimen Preparation

Twenty-five extracted human maxillary central incisors were selected after being radiographed buccolingually and mesiodistally. Inclusion criteria were as follows: single straight root canal, completely formed apex with patent foramina, no obstruction within canal system, and no evidence of internal and/or external resorption. Selected teeth were decoronated at 16 mm from the apex to standardize the length of all specimens. After pulp extirpation, size 10 K-file (Dentsply Maillefer) was introduced into the canal until it was visible at the apical foramen. True working length was established by subtracting 1.0 mm from this measurement.

Root canal preparation was carried out using the conventional multiple-file rotary ProTaper System (Dentsply Maillefer, Ballaigues, Switzerland) up to a master apical file size of F4 (size 40, 0.06). The canals were irrigated with 2.0 ml of 5.25% sodium hypochlorite after each filing. After completion of root canal preparation, 3.0 ml of 17% ethylenediaminetetraacetic acid (EDTA) (SmearClear™, SybronEndo, Orange, USA) was used to remove the smear layer. This was followed by rinsing with 3.0 ml of distilled water.

### 2.2. Obturation

Specimens were randomly divided into five groups according to the type of obturation materials and the roots were filled as follows:

Group 1: EndoREZ coated GP and EndoREZ sealer.

Group 2: Bioceramic coated GP with Endosequence BC sealer.

Group 3: Active GP with Endosequence BC sealer.

Group 4: Conventional GP (ISO color coded, Dentsply Maillefer) with Endosequence BC sealer.

Group 5: HAGP with Endosequence BC sealer.

Roots were obturated using a single matching cone obturation technique, without condensation to maintain experimental consistency among all groups. All groups were obturated with a sealer (applied according to manufacturer's instructions) and a single #40 0.06 tapered master cone. Due to the lack of #40 cones for the EndoREZ, the master GP cone #35 was customized for filling by sear off the cone from the apex using surgical blade and dipped in chloroform for 1 second at room temperature. The softened GP was placed in a dry canal to obtain the impression of apical seat. The cone was removed from the canal after 30 seconds and allowed to dry. When the EndoREZ filling was completed, the coronal surface was light cured with a light curing unit (Spectrum™ 800.Dentsply, Caulk, USA) for 40 seconds to produce an immediate coronal seal. All specimens were kept in an incubator at 37°C in 100% humidity for 10 days to allow sealers to set.

### 2.3. Push-Out Bond Strength

Each root was then embedded in cold-cure epoxy resin (Mirapox A and B; Miracon, Malaysia). After setting, the specimen was sectioned transversally using a water-cooled precision diamond saw (Metkon-Micracut 125 low speed precision cutter). The cutting disc was placed perpendicular to the long axis of the roots and the apical 3 mm of each root was discarded due to the small size of the filling material in this level. 5-6 successive root slices with 1 mm thickness were obtained from each root. Therefore, each group contained 25 root slices. For accuracy of calculation, each root slice thickness was verified using a digital caliper to within 0.01 mm (Mitutoyo/Digimatic, Tokyo, Japan).

**Figure 1:**
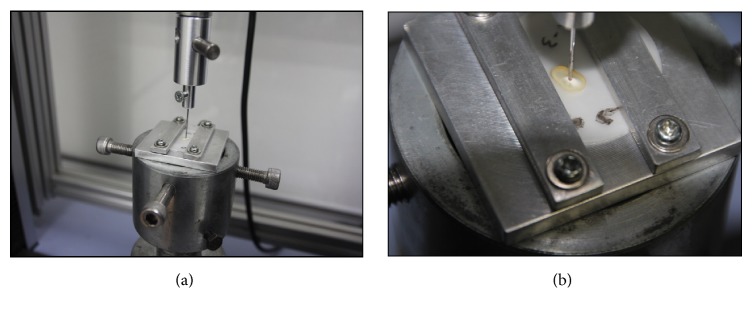
(a) Position and alignment of the specimen in the universal testing machine. (b) Only the root filling material was in contact with the plunger.

A Universal testing machine (Shimadzu, Japan) was equipped with a 0.8 mm diameter cylindrical stainless steel plunger. Each specimen was positioned in a customized fabricated jig to fix and align in a way that the apical surface faced the plunger. The plunger was in contact with the filling material only to avoid misreading by fracture the root dentin as shown in Figure 1. An increasing compressive load was applied to the filling material at a crosshead speed of 0.5 mm/min until bond failure occurred. The bond failure load (N) was recorded at the point where a sudden sharp drop of the stress-strain curve was displayed or complete dislodgement of the root filling material occurred. The bond strength in MPa was calculated by dividing the force (N) by the debonding area of the root canal filling (mm^2^). The bonded area was calculated using the following formula according to a previous study conducted by Sly et al. [14]:

Bonded area (mm^2^) = [Circumferential of apical aspect of root canal (mm) + Circumferential of coronal aspect of root canal (mm))/2] x thickness (mm).

### 2.4. Failure Analysis

After the push-out bond strength test, both sides of the specimens, including the main cone and sealer plugs, were examined under stereomicroscope (Leica SZ X7, Olympus, Japan) to determine the mode of failure. Each sample was evaluated at 56 x magnification and put into one of the categories according previous studies [15]: (i) adhesive failure either at the sealer/dentin or between the sealer/core interfaces at the, (ii) cohesive failure within the filling material, and (iii) mixed failure in both adhesive and cohesive modes.

### 2.5. Data Analysis

The data were subjected to statistical analysis in SPSS, version 12 (SPSS Inc., Chicago, USA). One-way parametric ANOVA test was used to analysis the mean push-out bond strength of various groups. The significant value was set at* p* = 0.05. Multiple comparisons post hoc Dunnett T3 test was used to detect the significant difference among the groups. Achi-square test (*p* = 0.05) was used to analyze the association between failure modes and compare between groups.

## 3. Results

**Figure 2:**
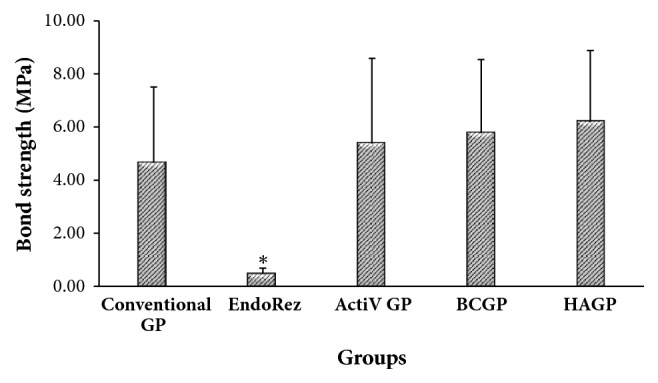
Mean push-out bond strength for all groups. *∗*Significant different *P* < 0.05.

The mean push-out bond strength for the various groups is shown in Figure 2. The highest bond strength was yielded by HAGP with a mean strength of 6.18 ± 2.70 MPa followed by BCGP (5.77 ± 2.78) MPa, ActiV GP (5.38 ± 3.21) MPa, conventional GP (4.65 ± 2.86) MPa, and EndoREZ (0.48 ± 0.20) MPa.

One-way analysis of variance (ANOVA) showed that there is significant difference among the various groups (*p* < 0.001). The Dunnett T3 post hoc showed that mean bond strength of EndoREZ group was significantly lesser than all other groups (*p* < 0.001). Even though the mean bond strength of HA GP group is higher than BC GP, ActiV GP, and conventional GP groups, no significant differences were detected among the other groups (*P* > 0.05). Chi square test showed significant difference among all groups. There is significant difference in failure mode between different types of root canal filling between (*p* =0.003). Failure mode is significantly associated with types of root canal filling (*p* =0.003). The prominent failure modes of conventional GP were cohesive and mixed (36%). EndoREZ exhibited higher adhesive failure mode (56%). HAGP showed high mixed failure mode (52%), whereas ActiV GP and BCGP showed cohesive failure, 48% and 44%, respectively.

## 4. Discussion

The thin-slice push-out test method is considered a reliable technique to measure the bond strength of root canal filling materials to root dentine [16]. The advantages of the thin-slice push-out test over tensile and shear strength tests are that it is less sensitive to small variations among specimens and to variations in stress distribution during load application, and it is easy to align samples for testing [17]. It also has been found reliable in bond strength evaluation in 1 mm-thick samples [18].

In this study, the sections from the middle level of the roots were used for analysis with same plunger size (0.8 mm) to reduce the variables that could affect the bond strength. Earlier studies report that the different sizes of the plunger used to push out the obturation material from different levels of the roots (apical, middle, and coronal) can influence the bond strength of the root sealer [19, 20]. Nevertheless, when the same plunger size is used for that purpose, the bond strength did not significantly vary between the root levels [8].

In this study, EndoREZ resulted in the lowest mean of push-out bond strength; this is in agreement with previous studies [15, 21]. Two factors could explain this finding: firstly, the polymerization shrinkage of the sealer and subsequent gap formation between sealer and canal wall [9]; secondly, the effect of cavity configuration factors (C-Factor). The C-Factor is found to be extremely high in long and narrow root canals. In these situations, there is insufficient unbonded surface area to provide relief from the stresses created by polymerization shrinkage resulting in the high risk of a pull-off or debonding at the interfaces [22]. Thirdly, it is due to the absence of an oxygen inhibition layer on the resin-coated GP that is removed to avoid the sticking of the cones during storage. This oxygen inhibition layer is necessary for optimal coupling of methacrylate-based resins of the resin sealer and resin-coated GP [23].

The higher bond strength for the other groups can be attributed to the nature of Endosequence BC sealer used. Endosequence BC is composed mainly of calcium silicate which uses the moisture naturally present in dentinal tubules to initiate and complete the setting reaction, hence no shrinkage occurs during setting [24]. Additionally, the nanofiller of this sealer can enhance the bond strength. As such, combining the nanosize fillers into the root sealers is advocated to help improve the bonding between the sealer and the root dentine [25].

Although ActiV GP, BCGP, HAGP, and conventional GP groups were used with the same sealer, they yielded different bond strength values. This can be an indicator for bonding behaviour of the coated materials to the sealer. It was reported that ActiV G has nonhomogeneous coating filler on the cones surface which may contribute to reduce bonding to the sealer [26].

It is well established that conventional GP cannot bond to root dentine as well as root sealers [4, 6] and this explained the lower bonding values of conventional GP group even with same sealer. BCGP yielded the second highest bond strength, which is in contrast to a previous study that stated that it had the lowest bond strength compared to GP/AH plus and experimental GP which contained a niobium phosphate glass composite [11]. This disagreement is due to the use of BCGP without any sealer in that study. HAGP yielded the highest push-out bond strength and this can be attributed to the components and roughness of the apatite calcium phosphate coating that increased the adhesion due to penetration of the sealer particles into the coating layer of the GP.

The failure mode of EndoREZ was mainly adhesive and this correlates with previous study [15]. This finding affirmed that adhesion of EndoREZ sealer to EndoREZ cones is stronger than to root dentin.

In the current study, cohesive failure mode was prominent in both ActiV GP and BC GP which indicates that the adhesion of Endosequence BC to root dentine is stronger than its adhesion to coated GP cones. This finding can be attributed to the coating thickness of both ActiV GP and BC GP which is around 2 *μ*m thickness. Additionally, the nonhomogeneous distribution of glass ionomer fillers on the surface of ActiV GP cones that were either devoid or dislodged can affect negatively the bond to the sealer [26].

The prominence of the mixed failure mode in the HAGP group was not unexpected and this is considered as evidence supporting the hypothesis in which the apatite coating for GP was used. The mixed failure mode can be an indicator of the equivalent bond strength of the Endosequence BC sealer to root dentine as well as to HAGP due to the similarity of the components in both the apatite calcium phosphate coating and the components of the root dentine.

## 5. Conclusion

HAGP showed promising results to be used as root canal filling material in combination with bioceramic sealer.

## Data Availability

The data used to support the findings of this study are available from the corresponding author upon request.
